# Breed-Specific Anaesthetic Mortality in Dogs: Evidence from an Analysis of 55,019 Cases

**DOI:** 10.3390/ani15213112

**Published:** 2025-10-27

**Authors:** José I. Redondo, Fernando Martínez-Taboada, Eva Zoe Hernández-Magaña, Luis Domenech, Jaime Viscasillas, Pablo E. Otero

**Affiliations:** 1 Departamento Medicina y Cirugía Animal, Facultad de Veterinaria, Universidad Cardenal Herrera—CEU, CEU Universities, 46115 Valencia, Spain; eva.hernandezmagana@uchceu.es; 2 Sydney School of Veterinary Science, University of Sydney, Sydney, NSW 2050, Australia; fer_m_taboada@hotmail.com; 3 Departamento de Matemáticas, Física y Ciencias Tecnológicas, Escuela Superior de Enseñanzas Técnicas, Universidad Cardenal Herrera—CEU, 46115 Valencia, Spain; luis.domenech@uchceu.es; 4 Anicura Valencia Sur Hospital Veterinario, Picassent 28, 46460 Silla Valencia, Spain; jaimeviscasillas2@gmail.com; 5 Department of Anaesthesiology and Pain Management, Facultad de Ciencias Veterinarias, Universidad de Buenos Aires, Buenos Aires C1427CWN CABA, Argentina; potero@fvet.uba.ar

**Keywords:** veterinary anaesthesia, anaesthetic mortality, dog, breed-specific risk, brachycephalic dogs, ABCB1, MDR1

## Abstract

Anaesthesia in dogs carries measurable risk, and whether this varies by breed is uncertain. We analysed a prospective, multicentre cohort of 55,019 canine anaesthetic procedures from premedication to 48 h post-extubation; after excluding euthanasias and non-anaesthetic deaths, 54,542 anaesthetics remained. Mixed-breed dogs served as the benchmark. Unadjusted mortality by breed, Fédération Cynologique Internationale (FCI) groups and sections used Wilson 95% CIs and χ^2^/Fisher tests; robust Poisson models adjusted for ASA status. Overall mortality was 0.69% (378/54,542; 95% CI 0.62–0.76), with mixed-breed dogs at 0.68% (109/16,129). Unadjusted rates were higher for German Shepherd Dog (1.46%), Chihuahua (1.35%) and Bulldog (1.26%); no FCI group differed, while the Chihuahueno section was higher. Brachycephalic dogs showed higher crude mortality than non-brachycephalics (0.82% vs. 0.65%), but this attenuated with ASA adjustment (RR 1.19, 95% CI 0.96–1.47). MDR1-associated breeds did not differ in crude or adjusted analyses (adjusted RR, 1.14; 95% CI, 0.61–2.14). After ASA adjustment, excess risk persisted for Chihuahua (RR 1.80, 95% CI 1.08–2.99) and Spanish Water Dog (RR 2.72, 95% CI 1.23–6.01), but not for German Shepherd Dog or Bulldog. Anaesthetic mortality was low overall; between-breed differences largely reflected case severity, supporting breed-aware, ASA-centred risk communication and further breed-specific research.

## Simple Summary

Owners often ask whether certain dog breeds face higher risks under anaesthesia. We analysed records from 55,019 anaesthetic procedures performed worldwide and tracked outcomes for up to two days after the operation. We excluded cases where dogs were put to sleep for other reasons or died from surgical or medical problems unrelated to anaesthesia. Overall, deaths linked to anaesthesia were rare (about 7 in 1000). Most breeds had risks similar to those of mixed-breed dogs. Three breeds showed higher figures before accounting for each dog’s health: German Shepherd Dog, Chihuahua, and Bulldog. Considering the health of the dogs before anaesthesia, only the Chihuahua and Spanish Water Dog still had elevated health indicators; the signals for the German Shepherd and Bulldog had not remained. Brachycephalic breeds seemed at higher risk, but this was mainly due to their health status, not their facial structure conformation. Breeds with a drug-sensitivity gene did not have higher death rates. These findings suggest that veterinarians should prioritise each dog’s overall health and provide extra airway care to flat-faced dogs while using breed information to facilitate transparent and honest discussions with owners. This evidence can help improve planning, consent, and safety for dogs needing anaesthesia.

## 1. Introduction

Anaesthesia is a key tool in small-animal practice. However, it remains associated with a measurable risk of adverse events and death. In the most recent global, prospective cohort of more than 55,000 canine anaesthetics, the overall anaesthesia-related mortality in dogs was 0.69% [1]. Despite advances in monitoring, multimodal analgesia and locoregional techniques, canine anaesthetic mortality remains orders of magnitude higher than contemporary estimates in human medicine [2]. 

Breed is a biologically plausible determinant of anaesthetic vulnerability through multiple pathways. For example, conformation-related airway compromise in brachycephalic breeds increases the likelihood of peri- and post-anaesthetic complications and amplifies the risk concentrated in recovery [3,4]. In parallel, pharmacogenetic variation in the ABCB1 (MDR1) gene alters P-glycoprotein function and can modulate sensitivity to several drugs relevant to peri-anaesthetic care; this variant shows an uneven breed distribution, especially among herding breeds and related lineages [5]. Beyond individual breeds, organisational classifications such as *Fédération Cynologique Internationale* (FCI) groups may proxy shared morphology and disease predispositions that could shape anaesthetic risk profiles [6].

However, robust, large-scale estimates of breed-specific anaesthesia-related mortality remain limited. Earlier landmark work quantified overall risk and identified key modifiers—higher ASA status, urgency, age and specific maintenance/induction strategies—yet did not primarily resolve how risk partitions by breed and related categories at scale [7,8,9,10,11]. The availability of an extensive, modern, multinational canine dataset with detailed breed recording [1] offers a timely opportunity to address this gap.

Our primary objective was to assess breed-specific risk, both before and after adjusting for pre-anaesthetic health status using the ASA classification, to distinguish genuine breed effects from case-mix influences. Simultaneously, we sought to place breeds within their broader taxonomic framework by summarising mortality across FCI groups and sections, and to illustrate the risk gradient across ASA classes within two pre-defined groups of interest: brachycephalic versus non-brachycephalic dogs, and breeds traditionally linked to the ABCB1 (MDR1) variant versus others. Additionally, we examined whether the impact of ASA differed across these groups. We hypothesised that (i) mortality would vary among individual breeds and FCI groups and sections compared to mixed-breed dogs; (ii) brachycephalic dogs and MDR1-associated breeds would each show higher anaesthesia-related mortality than their respective counterparts; and (iii) while ASA would remain a key predictor of outcome, these subgroup differences would persist, at least partly, after adjusting for ASA.

## 2. Materials and Methods

We performed a secondary analysis of a prospective, multicentre cohort of canine anaesthetics assembled across 405 participating centres in 21 countries on four continents, collected between 01/02/2016 and 31/12/2022 [1]. Spain (*n* = 29,517; 53.6%), Argentina (*n* = 11,555; 21.0%), France (*n* = 4560; 8.3%), the UK (*n* = 3139; 5.7%) and the USA (*n* = 2898; 5.3%) were the countries that sent the most cases. The parent cohort and data collection procedures were approved by the Ethics Committee of Universidad Cardenal Herrera-CEU (CEEA 22/07). The present analysis used de-identified records collected under that approval. Peri-anaesthetic data were recorded on a standardised case report form. The observation window extended from premedication to 48 h after extubation.

The source dataset in this paper contained 55,019 canine anaesthetic records, slightly lower than the 55,022 procedures reported in the parent study [1], owing to the subsequent removal of three empty entries during post hoc data cleaning. All dogs in the parent cohort that underwent general anaesthesia were eligible. The primary endpoint was anaesthesia-related death within the observation period. Deaths classified as euthanasia or cases where death was due to non-anaesthetic surgical or medical complications were excluded. Consequently, the analytic dataset comprised 54,542 dogs with a vital status of either alive or dead within 48 h.

The principal exposure was breed, recorded by the clinician. We further derived:

- FCI group and section. Breeds were mapped to the *Fédération Cynologique Internationale* (FCI) groups and sections [12].
- Brachycephalic conformation (yes/no). Defined a priori using a phenotype-based list grounded in the brachycephalic obstructive airway syndrome literature [3,4,6,13,14,15]. The following breeds in our dataset were classified as brachycephalic: Affenpinscher, American Bull Dog, American Cocker Spaniel, American Pit Bull Terrier, American Staffordshire Terrier, Boxer, Boston Terrier, Bull Arab, Bullmastiff, Bull Terrier, Brussels Griffon/Griffon Belge/Petit Brabançon, Bulldog, French Bulldog, Olde English Bulldogge, Italian Cane Corso, Cavalier King Charles Spaniel, Chihuahua, Chow Chow, Continental Bulldog, Dogue de Bordeaux, Dogo Argentino, English Mastiff, Japanese Chin, Lhasa Apso, Löwchen, Mastiff, Miniature Bull Terrier, Neapolitan Mastiff, Newfoundland, Pekingese, Presa Canario, Pug, Pyrenean Mastiff, Rottweiler, Shar Pei, Shih Tzu, Staffordshire Bull Terrier, Tibetan Spaniel, Tosa, and Yorkshire Terrier.
- MDR1-associated breeds (yes/no). A breed-level proxy for ABCB1-1Δ/nt230(del4) carriage based on published genotype surveys and case series [16]. The following breeds were considered potential carriers of this genetic mutation: Australian Shepherd, Black Mouth Cur, Bearded Collie, Border Collie, Chinook, Collie (Smooth and Rough), English Shepherd, Whippet, Old English Sheepdog, Shetland Sheepdog, Silken Windhound, Wäller, McNab, and White Swiss Shepherd Dog.

**Statistical analysis**

All analyses were carried out using R version 4.5.0. Data cleaning and visualisation were performed with tidyverse, importing data via the *readxl* package. Binomial confidence intervals (CIs) were calculated using the *binom* package. Robust inference was conducted with the *sandwich* and *lmtest* packages, and model outputs were organised with the *broom* package.

For the description, we counted alive and dead outcomes for each breed, FCI group, and FCI section, then calculated mortality proportions with 95% CIs using Wilson’s method. Each category was compared to the Mixed-breed benchmark using two-sided χ^2^ tests (without continuity correction) or Fisher’s exact test when any expected cell was <5. *p* values are nominal and unadjusted; they are reported to facilitate screening and should be considered exploratory. To visualize the screening results clearly, we created a volcano plot with the crude risk difference on the *x*-axis and the negative log10 of the *p*-value on the *y*-axis. This effectively highlights both the magnitude and the statistical significance of breed-specific differences.

For modelling, we estimated relative risks (RRs) with Poisson regression (log link) and heteroscedasticity-consistent (HC3) standard errors. We specified three parallel models: death~breed + ASA, death~FCI group + ASA, and death~FCI section + ASA, using Mixed Breed and ASA I as reference categories. For numerical stability and interpretability, the breed model was restricted to the 30 most prevalent breeds. In contrast, the FCI group and section models included all observed levels with complete covariate data. Analyses were performed on complete cases without imputation. To place ASA effects into a clinically meaningful context, we also refit models within brachycephalic (yes/no) and MDR1-associated (yes/no) strata, reporting RRs for ASA II–V versus ASA I. ASA × brachycephalic and ASA × MDR1 interactions were examined exploratorily only. We report RRs with robust 95% CIs and two-sided *p* values (α = 0.05).

## 3. Results

Of 55,019 canine anaesthetic procedures, 54,164 dogs survived, 378 experienced an anaesthesia-related death, 360 were euthanised, and 117 died from surgical or pre-existing disease. Excluding euthanasias and non-anaesthetic deaths yielded an analytic cohort of 54,542 anaesthetics. The anaesthesia-related mortality was 0.69% (378/54,542; 95% CI 0.62–0.76). Mixed-breed dogs were the largest category (n = 16,129) with an overall anaesthetic specific mortality of 0.68% (109/16,129; 95% CI 0.56–0.81).

**Figure 1:**
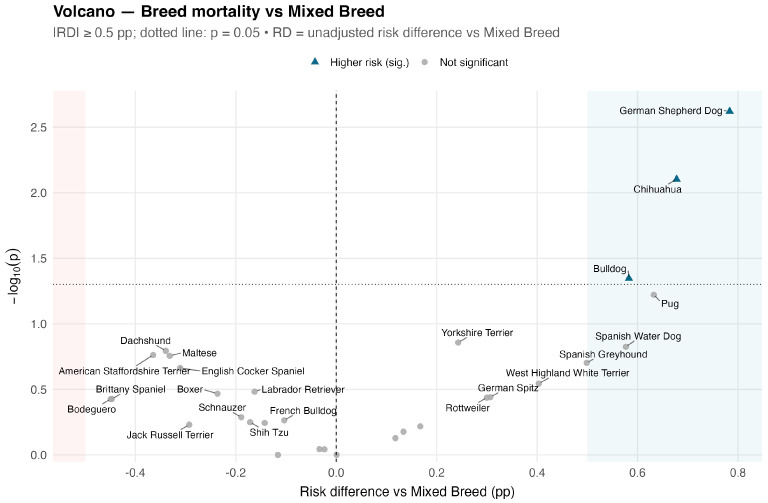
Volcano plot of unadjusted breed-specific differences in anaesthesia-related mortality vs. the Mixed-Breed reference (top 30 breeds by sample size).

Unadjusted mortality for the 30 most prevalent breeds is summarised in Table 1. At α = 0.05, three breeds exceeded the mixed-breed rate: German Shepherd Dog, 1.46% (17/1165; *p* = 0.002); Chihuahua, 1.35% (16/1182; *p* = 0.008); and Bulldog, 1.26% (11/874; *p* = 0.045). The volcano plot (Figure 1) displays unadjusted risk difference versus -log10(p): most breeds cluster near zero and below the significance threshold; the German Shepherd Dog and Chihuahua lie clearly above it, with Bulldog and Pug close to the boundary.

Including ASA in robust Poisson models attenuated most unadjusted breed signals. After adjustment, the increased risk persisted for the Chihuahua (RR 1.80, 95% CI 1.08–2.99; *p* = 0.023) and emerged for the Spanish Water Dog (RR 2.72, 95% CI 1.23–6.01; *p* = 0.013), which had shown no excess risk in the unadjusted comparison. Estimates for German Shepherd Dog (RR 1.47, 95% CI 0.88–2.44; *p* = 0.139) and Bulldog (RR 1.51, 95% CI 0.83–2.76; *p* = 0.177) were not significant after adjustment (Table 1). A borderline elevation was noted for the Spanish Greyhound (RR 2.01, 95% CI 1.00–4.06; *p* = 0.051).

**Figure 2:**
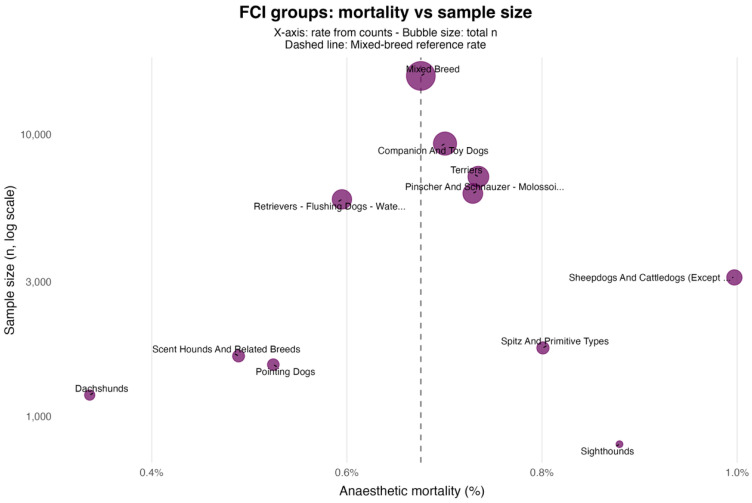
FCI groups: anaesthetic mortality vs. sample size (log scale). Each bubble represents an FCI group. The *x*-axis shows the crude anaesthetic mortality, estimated directly from counts (%), and the *y*-axis shows the group sample size on a logarithmic scale; the bubble area is proportional to the total sample size. The vertical dashed line marks the Mixed-breed reference rate. Values are unadjusted; statistical comparisons and Wilson 95% CIs are reported in Table 2.

**Figure 3:**
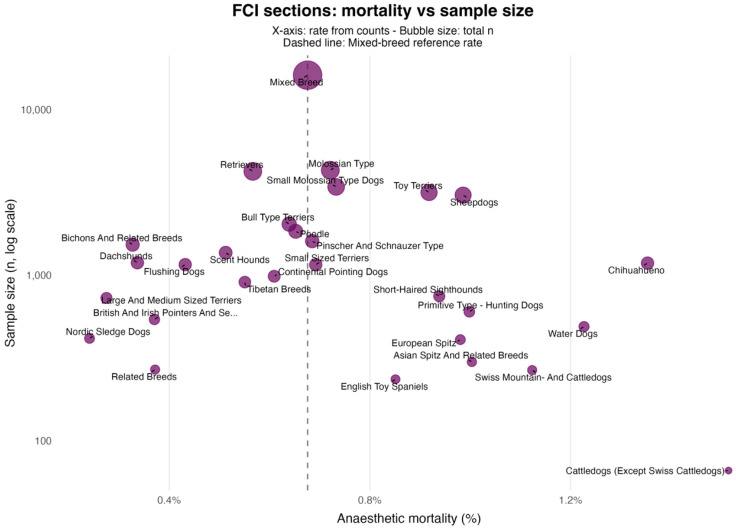
Each bubble denotes an FCI section. The *x*-axis shows the crude anaesthetic mortality from counts (%), and the *y*-axis shows the section sample size on a logarithmic scale; the bubble area scales with the total number. The vertical dashed line indicates the Mixed-breed reference rate. Results are unadjusted; corresponding *p*-values and Wilson 95% CIs are provided in Table 3.

Across *Fédération Cynologique Internationale* groups, unadjusted mortality rates ranged from 0.34% to 1.00% (Figure 2); none differed significantly from those of mixed-breed dogs (Table 2). At the section level, the Chihuahueno section (which includes only the Chihuahua breed) showed higher unadjusted mortality (1.35%; *p* = 0.008; Figure 3), whereas Sheepdogs approached but did not reach nominal significance (0.99%; *p* = 0.064) (Table 3).

Brachycephalic dogs had higher unadjusted mortality than non-brachycephalic dogs (0.82% [119/14,571] vs. 0.65% [259/39,971]; *p* = 0.036). This difference was attenuated after adjusting for ASA and was not statistically significant (adjusted RR, 1.19; 95% CI, 0.96–1.47; *p* = 0.112). MDR1-associated breeds did not differ from others on unadjusted (0.71% [10/1412] vs. 0.69% [368/53,130]; *p* = 0.945) or ASA-adjusted comparison (RR 1.14, 95% CI 0.61–2.14; *p* = 0.682). Full unadjusted and adjusted estimates are provided in Table 4.

## 4. Discussion

In this large, prospective, multicentre cohort of 55,019 canine anaesthetics, the overall anaesthesia-related mortality rate was relatively low (0.69%). Mixed-breed dogs—pre-specified as the benchmark—had a rate of 0.68%. Only a few common breeds showed higher unadjusted mortality (notably the Chihuahua, German Shepherd Dog and Bulldog). Most high-volume breeds were indistinguishable from the benchmark. After adjustment for ASA physical status using robust Poisson models, between-breed differences were markedly attenuated: an excess risk persisted for the Chihuahua and, emerging only after ASA adjustment, for the Spanish Water Dog. At the same time, estimates for the German Shepherd Dog and the Bulldog diminished and no longer reached conventional significance. These findings underscore the primacy of pre-anaesthetic health status in explaining observed variability, with any residual, breed-linked susceptibility confined to a small number of lineages.

These findings align well with the broader literature, which has consistently prioritised patient status and procedure over other factors. Landmark work from the Confidential Enquiry into Perioperative Small Animal Fatalities project and subsequent national cohorts identified higher ASA grade, urgency and major procedures as dominant predictors of anaesthetic death [7,8,9,10,11,17] with similar conclusions in a recent international analysis [1]. Our attenuation of unadjusted breed signals after ASA adjustment is consonant with that evidence base.

The biological plausibility of a brachycephalic signal is well established. Brachycephalic conformation compromises airway reserve and is associated with hypoventilation, dynamic obstruction and regurgitation around the peri-anaesthetic period [3,4,18]. Bulldogs are overrepresented among peri-anaesthetic complications, which are dominated by hypoxaemia and airway obstruction [19]. Meanwhile, reflux and aspiration are recognised sequelae of anaesthesia [20]. In toy breeds such as the Chihuahua, sedation and anaesthetic depth can precipitate airway collapse [21,22]. The small body size of these breeds increases the propensity for peri-anaesthetic hypothermia, a known risk factor [23,24]. Together, these mechanisms offer a coherent explanation for the persistence of excess risk in the Chihuahua after ASA adjustment.

By contrast, the higher unadjusted mortality observed in German Shepherd Dogs is plausibly explained by case-mix. This breed is over-represented among deep-chested dogs at risk of gastric dilatation–volvulus, an emergency presentation accompanied by hypovolaemia, metabolic derangements and a heavy burden of intra- and post-anaesthetic complications, including ventricular arrhythmias [25,26]. Since emergency status and higher ASA grade are themselves powerful drivers of mortality [1,7,8,10,11,17], attenuation of the German Shepherd Dog signal after ASA adjustment is unsurprising.

At broader taxonomic resolutions, unadjusted mortality across FCI groups ranged from 0.34% to 1.00%. In unadjusted two-by-two comparisons against the Mixed Breed benchmark, no group differed at α = 0.05. At the finer section level, only Chihuahueno—comprising the Chihuahua breed alone—exceeded the reference (*p* = 0.008), a finding that necessarily mirrors the breed-level result. This pattern is expected: aggregation within heterogeneous groups dilutes contrasts, whereas narrower sections can concentrate phenotype-linked risk. As our *p*-values are nominal (no formal multiplicity correction), section-level signals should be treated as exploratory and interpreted carefully. Confirmation in larger datasets with explicit control for multiple testing is warranted.

Two a priori strata further contextualise risk. First, brachycephalic dogs displayed higher unadjusted mortality than non-brachycephalic dogs, aligning with the pathophysiology of brachycephalic obstructive airway syndrome and reduced respiratory reserve across induction, maintenance and recovery [14,27,28]. This difference was materially reduced after adjustment for ASA, indicating that case severity explains a substantial share of the unadjusted contrast. Secondly, breeds historically associated with the ABCB1 nt230(del4) variant (MDR1) did not differ from others on unadjusted or ASA-adjusted comparison. This null is mechanistically coherent: ABCB1 loss-of-function increases sensitivity to specific substrates; under contemporary drug selection, dosing and monitoring, a cohort-level mortality excess is not inevitable [5]. Moreover, our MDR1 classification is a breed-level proxy rather than a genotype-confirmed one; carriage varies across breeds and geographies [29,30,31,32,33,34,35], so any actual effect would be diluted without individual genotyping or data on exposure to relevant substrates [36].

Methodologically, our approach combines transparent screening (two-by-two contrasts against a mixed-breed benchmark) with models that yield directly interpretable relative risks for uncommon outcomes (robust Poisson with HC3 errors). The volcano plot is helpful as a visual aid for triaging signals; however, extremes driven by sparse denominators—such as a single death in a small sample of a particular breed—should not be overinterpreted. Wilson intervals provide stable uncertainty quantification at low event rates, and the consistent emergence of ASA as the dominant prognostic factor mirrors prior veterinary studies [1,7,8,9,10,11,17].

These results have pragmatic implications. For most breeds, the absolute risk is low and closely aligned with that of mixed-breed dogs; breed alone should seldom determine anaesthetic decision-making. For brachycephalic patients, airway-centred planning remains warranted: experienced intubation, readiness for a difficult airway, protective strategies against reflux/aspiration, active thermal management, cautious extubation and vigilant recovery observation [4,13]. MDR1-associated status at the breed level is an imperfect surrogate; risk mitigation is better anchored in judicious drug choice and dose, and targeted genotyping when exposure to ABCB1 substrates is anticipated [5].

Several limitations should be noted in our study. First, clinicians reported breed and were therefore susceptible to misclassification—especially in visually similar or designer crossbreeds. Non-differential error would likely weaken true differences between breeds, while differential error across centres could bias the results in either direction. Second, brachycephalic and MDR1 statuses were assigned at the breed level; however, craniofacial morphology and ABCB1 genotype vary within breeds [5,14,28], meaning individual risk may be misrepresented and any genuine effects diminished. Third, attributing outcomes is inherently difficult: distinguishing an “anaesthesia-related” death from surgical or disease causes is imperfect, and recent evidence shows only moderate agreement among anaesthetists [37]. Such misclassification could reduce or distort breed differences, especially if attribution is linked to case-mix. Fourth, residual confounding is still possible. Many comparisons are unadjusted or only adjust for ASA; we lacked standardised data on urgency, procedure type, centre protocols, airway interventions, drug exposures (including ABCB1 substrates), and peri-operative management. Additionally, our models did not account for clustering by centre, so unmeasured site-level differences could inflate precision or bias estimates. Fifth, to maintain numerical stability, we limited adjusted models to the 30 most common breeds; signals in rarer breeds may therefore be overlooked, and small sample sizes in some groups lead to wide CIs and unstable estimates. Finally, multiple hypothesis testing across numerous breeds and taxonomic levels increases the risk of false-positive results; these should be presented with standard α thresholds and interpreted cautiously.

## 5. Conclusions

In summary, canine anaesthesia-related mortality in contemporary practice is low. Much of the between-breed variability is explained by pre-anaesthetic status, as captured by the ASA classification; yet a small number of breeds—notably the Chihuahua and the Spanish Water Dog—retain evidence of increased risk after adjustment. Brachycephalic conformation showed a modest, unadjusted excess that attenuated with ASA adjustment, whereas MDR1-associated status was not associated with mortality. These findings support breed-aware but ASA-centred risk communication, with airway-focused planning for brachycephalic patients. Future studies that incorporate procedure type and urgency, airway and thermal management, drug exposures (including ABCB1 substrates), centre effects, formal control of multiple comparisons, and individual phenotyping/genotyping will strengthen causal inference and refine patient-specific counselling.

## Figures and Tables

**Table 1:** Anaesthetic mortality by breed (top 30). Unadjusted *p*-values from χ^2^/Fisher using Mixed Breed as reference; adjusted RRs from robust Poisson (BREED + ASA).

Breed	Total	Deaths	Unadj. Mortality (%)	95% CI	*p* (Unadj.)	Adj. RR (ASA)	Adj. RR 95% CI	*p* (Adj., ASA)
Mixed Breed	16,129	109	0.68	0.56–0.81	-	1.00	-	-
Yorkshire Terrier	3157	29	0.92	0.64–1.32	0.139	1.24	0.84–1.84	0.286
Labrador Retriever	2728	14	0.51	0.31–0.86	0.329	0.64	0.37–1.12	0.116
French Bulldog	2621	15	0.57	0.35–0.94	0.544	0.85	0.50–1.46	0.556
Poodle	1840	12	0.65	0.37–1.14	0.907	1.01	0.56–1.82	0.984
Golden Retriever	1478	10	0.68	0.37–1.24	0.997	0.85	0.45–1.60	0.604
Dachshund	1189	4	0.34	0.13–0.86	0.161	0.55	0.21–1.45	0.229
Chihuahua	1182	16	1.35	0.83–2.19	0.008	1.80	1.08–2.99	0.023
German Shepherd Dog	1165	17	1.46	0.91–2.32	0.002	1.47	0.88–2.44	0.139
Maltese	1162	4	0.34	0.13–0.88	0.175	0.59	0.22–1.59	0.300
Boxer	1138	5	0.44	0.19–1.02	0.341	0.49	0.20–1.20	0.119
Beagle	1125	6	0.53	0.24–1.16	0.570	0.89	0.40–1.96	0.770
English Cocker Spaniel	1096	4	0.36	0.14–0.93	0.217	0.50	0.19–1.35	0.172
American Staffordshire Terrier	964	3	0.31	0.11–0.91	0.173	0.49	0.16–1.47	0.201
Border Collie	934	6	0.64	0.29–1.39	0.903	1.04	0.46–2.37	0.928
Bulldog	874	11	1.26	0.70–2.24	0.045	1.51	0.83–2.76	0.177
Schnauzer	822	4	0.49	0.19–1.24	0.516	0.59	0.22–1.59	0.294
Shih Tzu	793	4	0.50	0.20–1.29	0.563	0.70	0.26–1.92	0.492
American Pit Bull Terrier	741	6	0.81	0.37–1.76	0.665	1.12	0.50–2.54	0.782
Pug	688	9	1.31	0.69–2.47	0.060	1.85	0.94–3.63	0.074
Spanish Greyhound	596	7	1.17	0.57–2.40	0.198	2.01	1.00–4.06	0.051
Ibizan Hound	593	5	0.84	0.36–1.96	0.605	1.48	0.63–3.48	0.368
West Highland White Terrier	556	6	1.08	0.50–2.33	0.286	1.51	0.71–3.23	0.289
Miniature Pinscher	536	3	0.56	0.19–1.63	1.000	0.94	0.31–2.86	0.907
Jack Russell Terrier	522	2	0.38	0.11–1.39	0.588	0.66	0.17–2.56	0.543
Spanish Water Dog	479	6	1.25	0.58–2.71	0.150	2.72	1.23–6.01	0.013
Bodeguero	441	1	0.23	0.04–1.27	0.375	0.39	0.05–2.88	0.358
Brittany Spaniel	437	1	0.23	0.04–1.28	0.375	0.28	0.04–2.04	0.208
Rottweiler	410	4	0.98	0.38–2.48	0.366	1.09	0.40–2.94	0.871
German Spitz	407	4	0.98	0.38–2.50	0.362	1.34	0.50–3.59	0.562

Unadjusted mortality is presented as a percentage of anaesthetic procedures ending in death, with Wilson 95% CIs. Relative risks (RRs) estimated using robust Poisson regression, adjusted for ASA physical status.

**Table 2:** Anaesthetic mortality by FCI group. Mixed Breed is the reference. Adjusted *p*-values from χ^2^/Fisher; adjusted RRs from robust Poisson (FCI group + ASA).

Group	Total	Deaths	Mortality (%)	95% CI	*p* (Unadj.)	Adj. RR (ASA)	Adj. RR 95% CI	*p* (Adj, ASA)
Mixed Breed	16,129	109	0.68	0.56–0.81	-	1.00	-	-
Companion and Toy Dogs	9278	65	0.70	0.55–0.89	0.818	1.02	0.76–1.38	0.878
Terriers	7077	52	0.73	0.56–0.96	0.618	1.04	0.76–1.43	0.806
Pinscher and Schnauzer—Molossoid and Swiss Mountain and Cattle Dogs	6172	45	0.73	0.55–0.97	0.667	0.85	0.60–1.19	0.347
Retrievers—Flushing Dogs—Water Dogs	5883	35	0.59	0.43–0.83	0.510	0.78	0.53–1.13	0.188
Sheepdogs and Cattle Dogs (Except Swiss Cattle Dogs)	3109	31	1.00	0.70–1.41	0.054	1.24	0.84–1.83	0.286
Spitz and Primitive Types	1748	14	0.80	0.48–1.34	0.548	1.14	0.67–1.93	0.633
Scent Hounds and Related Breeds	1636	8	0.49	0.25–0.96	0.373	0.71	0.35–1.43	0.333
Pointing Dogs	1525	8	0.52	0.27–1.03	0.487	0.59	0.29–1.21	0.149
Dachshunds	1189	4	0.34	0.13–0.86	0.161	0.55	0.21–1.45	0.224
Sighthounds	796	7	0.88	0.43–1.80	0.497	1.48	0.72–3.03	0.288
Total (all groups)	54,542	378	0.69	0.62–0.76	-	-	-	-

Unadjusted mortality presented as % of anaesthetic procedures ending in death, with Wilson 95% CIs. Relative risks (RRs) estimated using robust Poisson regression, adjusted for ASA physical status.

**Table 3:** Anaesthetic mortality by FCI section. Mixed Breed is the reference. Unadjusted *p*-values from χ^2^/Fisher; adjusted RRs from robust Poisson (FCI section + ASA).

Section	Total	Deaths	Mortality (%)	95% CI	*p* (Unadj.)	Adj. RR (ASA)	Adj. RR 95% CI	*p* (Adj, ASA)
Mixed Breed	16,129	109	0.68	0.56–0.81		1.00		
Molossian Type	4299	31	0.72	0.51–1.02	0.749	0.81	0.55–1.19	0.283
Retrievers	4236	24	0.57	0.38–0.84	0.432	0.71	0.46–1.10	0.122
Small Molossian Type Dogs	3412	25	0.73	0.50–1.08	0.714	1.08	0.70–1.66	0.738
Toy Terriers	3159	29	0.92	0.64–1.32	0.140	1.23	0.83–1.83	0.295
Sheepdogs	3043	30	0.99	0.69–1.40	0.064	1.22	0.82–1.81	0.334
Bull Type Terriers	2034	13	0.64	0.37–1.09	0.849	0.88	0.50–1.54	0.652
Poodle	1840	12	0.65	0.37–1.14	0.907	1.00	0.55–1.81	0.997
Pinscher and Schnauzer Type	1606	11	0.68	0.38–1.22	0.966	0.88	0.48–1.61	0.680
Bichons and Related Breeds	1529	5	0.33	0.14–0.76	0.104	0.53	0.22–1.28	0.157
Scent Hounds	1366	7	0.51	0.25–1.05	0.475	0.79	0.38–1.67	0.539
Dachshunds	1189	4	0.34	0.13–0.86	0.161	0.55	0.21–1.45	0.224
Chihuahueno	1182	16	1.35	0.83–2.19	0.008	1.80	1.08–2.99	0.024
Flushing Dogs	1158	5	0.43	0.18–1.01	0.322	0.58	0.24–1.40	0.224
Small Sized Terriers	1156	8	0.69	0.35–1.36	0.948	1.05	0.54–2.06	0.882
Continental Pointing Dogs	985	6	0.61	0.28–1.32	0.804	0.65	0.29–1.49	0.311
Tibetan Breeds	908	5	0.55	0.24–1.28	0.653	0.79	0.32–1.94	0.605
Short-Haired Sighthounds	746	7	0.94	0.46–1.92	0.396	1.60	0.78–3.28	0.196
Large and Medium Sized Terriers	728	2	0.27	0.08–1.00	0.244	0.49	0.12–1.99	0.318
Primitive Type—Hunting Dogs	601	6	1.00	0.46–2.16	0.311	1.63	0.77–3.45	0.205
British and Irish Pointers and Setters	540	2	0.37	0.10–1.34	0.589	0.46	0.11–1.87	0.275
Water Dogs	489	6	1.23	0.56–2.65	0.156	2.42	1.07–5.46	0.033
Nordic Sledge Dogs	415	1	0.24	0.04–1.35	0.531	0.31	0.04–2.27	0.249
European Spitz	408	4	0.98	0.38–2.49	0.363	1.33	0.50–3.56	0.570
Asian Spitz and Related Breeds	299	3	1.00	0.34–2.91	0.461	1.31	0.44–3.90	0.627
Related Breeds	269	1	0.37	0.07–2.08	1.000	0.40	0.05–2.96	0.373
Swiss Mountain- and Cattle Dogs	267	3	1.12	0.38–3.25	0.433	1.45	0.48–4.40	0.515
English Toy Spaniels	235	2	0.85	0.23–3.05	0.675	0.83	0.20–3.41	0.792
Japan Chin and Pekingese	70	0	0.00	0.00–5.20	1.000	0.00	0.00–0.00	<0.001
Cattle Dogs (Except Swiss Cattle Dogs)	66	1	1.52	0.27–8.10	0.363	2.51	0.33–19.13	0.375
Continental Toy Spaniel and Others	58	0	0.00	0.00–6.21	1.000	0.00	0.00–0.00	<0.001
Long-Haired or Fringed Sighthounds	37	0	0.00	0.00–9.41	1.000	0.00	0.00–0.00	<0.001
Hairless Dogs	22	0	0.00	0.00–14.87	1.000	0.00	0.00–0.00	<0.001
Small Belgian Dogs	22	0	0.00	0.00–14.87	1.000	0.00	0.00–0.00	<0.001
Rough-Haired Sighthounds	13	0	0.00	0.00–22.81	1.000	0.00	0.00–0.00	<0.001
Nordic Watchdogs and Herders	10	0	0.00	0.00–27.75	1.000	0.00	0.00–0.00	<0.001
Primitive Type	10	0	0.00	0.00–27.75	1.000	0.00	0.00–0.00	<0.001
Nordic Hunting Dogs	5	0	0.00	0.00–43.45	1.000	0.00	0.00–0.00	<0.001
Leash (Scent) Hounds	1	0	0.00	0.00–79.35	1.000	0.00	0.00–Inf	1.000
Total (all groups)	54,542	378	0.69	0.62–0.76	-	-	-	-

Unadjusted mortality presented as % of anaesthetic procedures ending in death, with Wilson 95% CIs. Relative risks (RRs) estimated using robust Poisson regression, adjusted for ASA physical status.

**Table 4:** Anaesthetic mortality by ASA status, stratified by brachycephalic and MDR1 grouping.

Breed	Total	Deaths	Unadjusted Mortality (%)	95% CI	*p* (Unadjusted)	Adj. RR (ASA)	Adj. RR 95% CI	*p* (Adj, ASA)
Non-brachycephalic	39,971	259	0.65	0.57–0.73	-	1.00	-	-
Brachycephalic	14,571	119	0.82	0.68–0.98	0.036	1.19	0.96–1.47	0.112
Non-MDR1	53,130	368	0.69	0.63–0.77		1.00		
MDR1	1412	10	0.71	0.39–1.30	0.944	1.14	0.61–2.14	0.682
Total (all groups)	54,542	378	0.69	0.62–0.76	-	-	-	-

Unadjusted mortality presented as % of anaesthetic procedures ending in death, with Wilson 95% CIs. Relative risks (RRs) estimated using robust Poisson regression, adjusted for ASA physical status.

## Data Availability

The data that support the findings of this study are available from the corresponding author upon reasonable request.

## Abbreviations

The following abbreviations are used in this manuscript:

Abbreviation	Meaning
ABCB1	ATP-binding cassette sub-family B member 1 (P-glycoprotein) gene
ASA	American Society of Anesthesiologists physical status classification
CEEA	Comité de Ética en Experimentación Animal (Ethics Committee)
CIs	Confidence intervals
FCI	Fédération Cynologique Internationale
HC3	Heteroscedasticity-consistent covariance estimator, type 3
MDR1	Multidrug resistance gene 1 (historical name for ABCB1)
nt230(del4)	Four-base-pair deletion in ABCB1 (also written ABCB1-1Δ)
RR	Relative risk
